# Time-resolved fluoroimmunoassay for bactericidal/permeability-increasing protein

**DOI:** 10.1155/S0962935196000087

**Published:** 1996-02

**Authors:** J.-O. Häggblom, A. B. Jokilammi-Siltanen, H. Peuravuori, T. J. Nevalainen

**Affiliations:** 1 Department of Pathology University of Turku Kiinanmyllynkatu 10 Turku FIN-20520 Finland

## Abstract

Bactericidal/permeability-increasing protein (BPI) is a cationic antimicrobial protein produced by polymorphonuclear leukocytes, that specifically interacts with and kills Gram-negative bacteria. BPl competes with lipopolysaccharide-binding protein (LBP) secreted by liver cells into blood plasma for binding to lipopolysaccharide (LPS) and thus reduces the proinflammatory effects of LPS. We have developed a time-resolved fluoroimmunoassay for BPI and measured the concentration of BPI in human serum and plasma samples. The assay is based on a rabbit antibody against recombinant BPI. This antibody specifically adheres to polymorphonuclear leukocytes in immunostained human tissues. The difference in the serum concentration of BPI between unselected hospitalized patients with and without an infection was statistically significant. The mean concentration of BPI in serum samples was 28.3 μg/l (range 1.64–132, S.D. 26.8, *n* = 83). In contrast, there was no difference between the two groups in the BPI levels in plasma samples. For all individuals tested, BPI levels were consistently higher in plasma samples compared to the matched serum samples. The mean concentration of BPI in plasma samples was 52.3 μg/l (range 0.9–403, S.D. 60.6, *n* = 90). There was a positive correlation between the concentration of BPI and the white blood cell count as well as between the BPI concentration and C-reactive protein (CRP) in serum samples. In conclusion, the present study demonstrates that BPI can be quantified reliably by time-resolved fluoroimmunoassay in human serum samples.


Mediators of Inflammation 5, 47–50 (1996)

### Time-resolved fluoroimmunoassay for bactericidal/permeability-increasing protein

J.-O. Häggblom, A. B. Jokilammi-Siltanen, H. Peuravuori and T. J. Nevalainen[CA]

Department of Pathology, University of Turku, Kiinanmyllynkatu 10, FIN-20520, Turku, Finland. Fax: (+358) 21 6337459.

[CA]Corresponding Author

**BACTERICIDAL/permeability-increasing protein (BPI) is a cationic antimicrobial protein produced by polymorphonuclear leukocytes, that specifically interacts with and kills Gram-negative bacteria. BPI competes with lipopolysaccharide-binding protein (LBP) secreted by liver cells into blood plasma for binding to lipopolysaccharide (LPS) and thus reduces the proinflammatory effects of LPS. We have developed a time-resolved fluoroimmunoassay for BPI and measured the concentration of BPI in human serum and plasma samples. The assay is based on a rabbit antibody against recombinant BPI. This antibody specifically adheres to polymorphonuclear leukocytes in immunostained human tissues. The difference in the serum concentration of BPI between unselected hospitalized patients with and without an infection was statistically significant. The mean concentration of BPI in serum samples was 28.3 μg/l (range 1.64–132, S.D. 26.8, $n = 83$). In contrast, there was no difference between the two groups in the BPI levels in plasma samples. For all individuals tested, BPI levels were consistently higher in plasma samples compared to the matched serum samples. The mean concentration of BPI in plasma samples was 52.3 μg/l (range 0.9–403, S.D. 60.6, $n = 90$). There was a positive correlation between the concentration of BPI and the white blood cell count as well as between the BPI concentration and C-reactive protein (CRP) in serum samples. In conclusion, the present study demonstrates that BPI can be quantified reliably by time-resolved fluoroimmunoassay in human serum samples.**

**Key words:** Bactericidal/permeability-increasing protein, ELISA, Polymorphonuclear leukocytes, Serum/plasma protein, Time-resolved fluoroimmunoassay

### Introduction

The bactericidal/permeability-increasing protein (BPI) is a cationic antimicrobial protein produced by polymorphonuclear leukocytes. BPI specifically interacts with and kills Gram-negative bacteria. BPI binds to the lipopolysaccharide (LPS) component of the outer membrane of Gram-negative bacteria, increases membrane permeability to hydrophobic substances and causes irreversible loss of bacterial cell homeostasis.[1–6] BPI competes with lipopolysaccharide-binding protein (LBP) secreted by liver cells into the blood plasma for binding to LPS.[7–10] In this way BPI reduces the proinflammatory effects of LPS.[11–19]

Because there is potential anti-infectious therapeutic use for recombinant BPI,[20–25] a sensitive assay which can measure BPI in body fluids is needed. The purpose of the present study was to develop a time-resolved fluoroimmunoassay (TR-FIA) for the measurement of the concentration of BPI in human serum.

### Materials and Methods

*Instrumentation:* Time-resolved fluorescence was measured with an Arcus fluorometer (Wallac, Turku, Finland). The plate washer (Wellwash) and plate shaker (Delfia Plateshake) used in the fluoroimmunoassay were from Denley (Billinghurst, England) and Wallac (Turku, Finland), respectively. Data were handled with MultiCalc data management software (Wallac, Turku, Finland).

*Serum and plasma samples:* Serum and plasma samples were collected from unselected hospital-

© 1996 Rapid Science Publishers ized patients with and without an infection (42 women and 48 men). The average age was 61 years (range 14–93 years). Samples were stored frozen at −20°C until assayed.

*BPI standards:* The BPI cDNA was cloned and expressed in a Chinese hamster ovary cell line as described elsewhere.[26] BPI standards were prepared from recombinant human BPI (kindly donated by Dr Marian Marra, Incyte Pharmaceuticals, Inc., Palo Alto, CA, USA) stock solution into assay buffer (Wallac, Turku, Finland) to give five concentrations (4.07, 9.76, 48.8, 122 and 305 μg/l).

*Preparation of antibodies to recombinant human BPI:* Antiserum to recombinant BPI was raised in a rabbit. The rabbit was immunized four times at 3-week intervals subcutaneously with 0.05–0.2 mg of human recombinant BPI (Incyte, Palo Alto, CA, USA) in Freund's complete adjuvant at the first immunization and in Freund's incomplete adjuvant on later occasions. Serum was collected 2 weeks after the last booster injection.

*Labelling of anti-BPI antibody:* Protein A-purified anti-recombinant BPI antibody was labelled with an isothiocyanate derivative of a europium chelate ($Eu^{3+}$-*N*-(*p*-isothiocyanatobenzyl)-diethylene-tri-amine-$N^1$,$N^2$,$N^3$,$N^3$-tetra-acetate) by using an Eu-labelling kit (Wallac, Turku, Finland) according to the manufacturer's instructions.

*Time-resolved fluoroimmunoassays:* For the TR-FIA, microtitre plates were coated overnight with protein A-purified anti-BPI antibody (25 μg/ml in 50 mmol/l Tris-HCl, pH 7.75/0.15 mmol/l NaCl/ 0.05% $NaN_3$, 200 μl/well) treated with three volumes of HCl/water (125 μl of 11.6 M HCl in 50 ml of water) for 5 min. Coated plates were washed two times and 25 μl of BPI standard (0, 4.07, 9.76, 48.8, 122 and 305 μg/l) or sample were pipetted into the wells containing 175 μl of assay buffer. After 1 h incubation, with shaking, at room temperature and washing six times, Eu-labelled anti-BPI antibody (2.5 μg/ml in assay buffer, 200 μl/well) was added. The washing step was repeated after 1 h and 200 μl of enhancement solution (Wallac, Turku, Finland) was added. Fluorescence was measured after a further 5 min shaking and 10 min standing. Microtitre plates were from Eflab (Helsinki, Finland) and assay buffer for TR-FIA was from Wallac (Turku, Finland).

*Immunostaining:* Sections of formalin-fixed, paraffin-embedded human tissues from the files of the Department of Pathology, University of Turku were reacted with an IgG fraction of polyclonal rabbit anti-BPI antiserum, and the primary immunoreaction was localized as described previously[27] by using a Vectastain ABC kit (Vector Laboratories, Burlinghame, CA, USA) according to manufacturer's instructions. The intensity of immunostaining improved when the sections were heated for 2 × 5 min in a microwave oven before staining. For controls, the primary antibody was replaced by preimmune rabbit serum. The sections were counterstained by haematoxylin.

*Statistical analysis:* Student's *t*-test and Pearson's linear regression were used for statistical analysis.

### Results

The mean concentration of BPI in plasma ($n = 90$) was 52.3 μg/l (range 0.9–403, S.D. 60.6) and in serum samples ($n = 83$) 28.3 μg/l (range 1.64–132, S.D. 26.8). The linear range for the BPI standard curve was 5–500 μg/l (Fig. 1). The detection limit of the assay was 1.6 μg/l corresponding to the mean ± 3 S.D. of the zero standard (blank) fluorescence counts. The difference in the serum concentration of BPI between unselected hospitalized patients with and without infection was statistically significant ($p < 0.0001$, Fig. 2). The mean concentration of BPI in serum samples for all measured patients was 28.3 μg/l (range 1.64–132, S.D. 26.8, $n = 83$). In contrast, there was no difference between the two groups in plasma samples. For all individuals tested, BPI

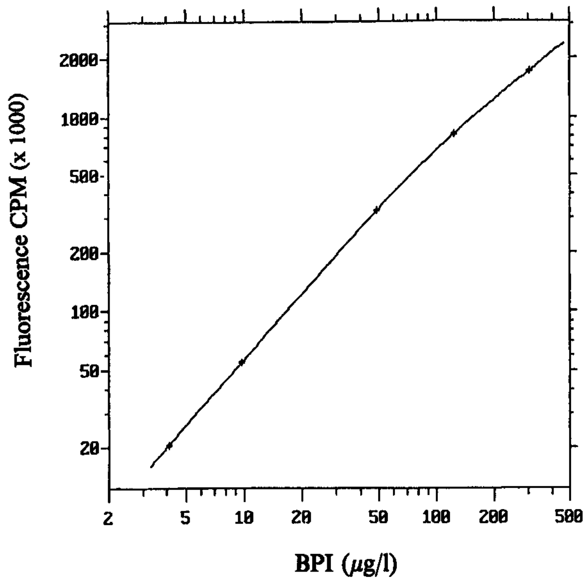

FIG. 1. The standard curve of time-resolved fluoroimmunoassay for BPI three separate assays.

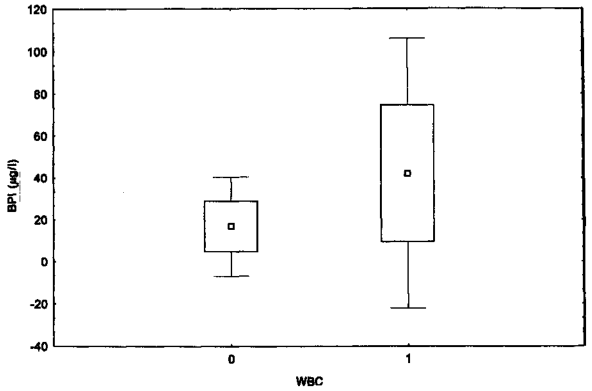

FIG. 2. Difference in the BPI concentrations between unselected hospitalized patients without an infection (group 0, $n = 45$) and patients with an infection (group 1, $n = 38$, $p < 0.0001$). Student's *t*-test was used for statistical analysis.

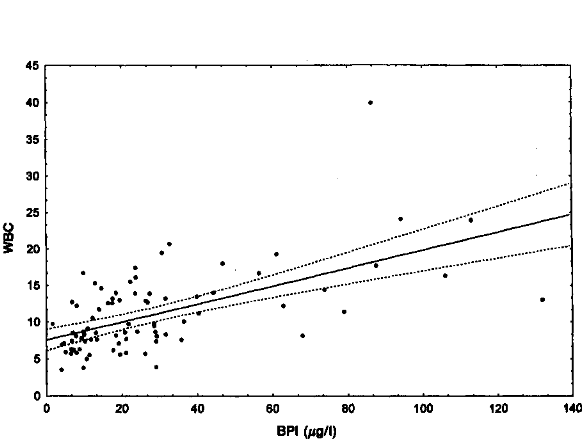

FIG. 3. Linear regression with 95% confidention intervals between blood white cell count (WBC, $\times 10^9$/l) and serum BPI concentration ($r = 0.589$, $p < 0.0001$, $n = 83$).

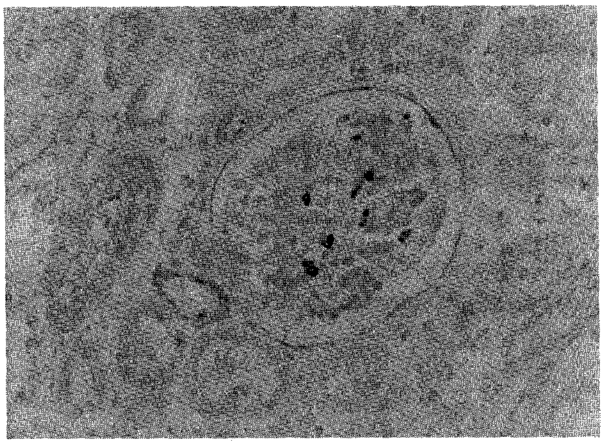

FIG. 4. Immunoreaction for BPI in polymorphonuclear leukocytes in the vascular compartment of a glomerulus of human kidney. Anti-BPI antibody. Avidin-biotin-peroxidase complex, haematoxylin counterstaining. Magnification 330×.

levels were consistently higher in plasma samples compared to the matched serum samples. The mean concentration of BPI in plasma samples was 52.3 µg/l (range 0.9–403, S.D. 60.6, $n = 90$).

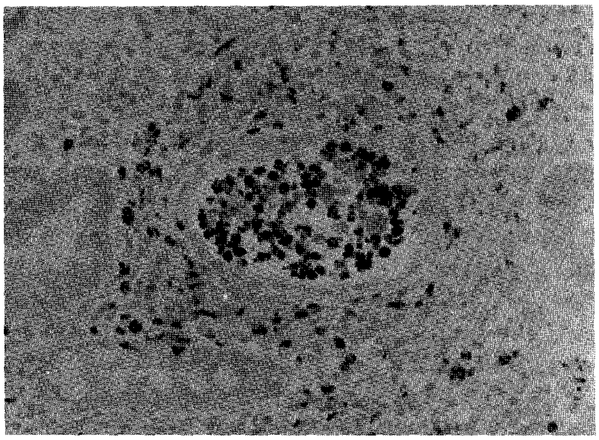

FIG. 5. Immunoreaction for BPI in polymorphonuclear leukocytes in the vascular compartment of colonic mucosa. Anti-BPI antibody. Avidin-biotin-peroxidase complex, haematoxylin counterstaining. Magnification 330×.

There was a positive correlation between the concentration of BPI in serum and the white blood cell count ($r = 0.589$, $p < 0.0001$, $n = 83$) (Fig. 3). There was also a positive correlation between serum BPI and CRP levels ($r = 0.39$, $p < 0.05$, $n = 59$). However, in plasma samples there was no correlation between BPI and white blood cell count or CRP. Intense immunoreaction was seen in polymorphonuclear leukocytes at numerous locations, e.g. in the vascular compartment of kidney glomeruli (Fig. 4) and colonic mucosa (Fig. 5). Control sections reacted with preimmune serum were devoid of immunoreaction.

### Discussion

The present paper describes a new immunoassay using time-resolved fluorescence technology for measuring the concentration of BPI. An enzyme immunoassay (ELISA) for determining the concentration of BPI was recently developed by White and coworkers.[28] The mean concentration of BPI in serum as measured by the ELISA[28] and the current TR-FIA are very similar (27.1 µg/l and 28.3 µg/l, respectively). As determined by the current TR-FIA, there was a positive correlation between the concentration of BPI and the white blood cell count as well as between the serum BPI and CRP values. A statistically significant difference was found in the serum BPI levels between patients with and without manifest infections by the current TR-FIA. Furthermore, the presence of BPI in polymorphonuclear leukocytes was confirmed by immunohistochemistry in the current study. Thus, the serum concentration of BPI seems to reflect the intensity of the inflammatory process in the body.

The mean concentration of BPI in heparinized plasma samples was markedly higher than that in serum samples as determined by the current assay. However, no correlations was found between BPI values and white blood cell counts in plasma samples. Furthermore, BPI concentrations varied randomly in plasma samples when ammonium-heparin, sodium citrate and EDTA were used as anticoagulants in preliminary tests (data not shown). Thus, the detection of BPI in plasma calls for further studies.

In conclusion, the concentration of BPI can be measured reliably in human serum by time-resolved fluoroimmunoassay.

### References

1. Gray PW, Flaggs G, Leong SR, Gumina RJ, Weiss J, Ooi CE, Elsbach P. Cloning of a human neutrophil bactericidal protein. *J Biol Chem* 1989; **264:** 9505–9509.
2. Ooi CE, Weiss J, Elsbach P, Frangione B, Mannion B. A 25-kDa $NH_2$-terminal fragment carries all the antibacterial activities of the human neutrophil 60-kDa bactericidal permeability-increasing protein. *J Biol Chem* 1987; **262:** 14891–14894.
3. Elsbach P, Weiss J. Phagocytosis of bacteria and phospholipid degradation. *Biochim Biophys Acta* 1988; **947:** 29–52.
4. Weiss J, Elsbach P, Shu C, Castillo J, Grinman J, Horwitz A, Theofan G. The human bactericidal/permeability-increasing protein and a recombinant NH2-terminal fragment cause killing of serum resistant gram-negative bacteria. *J Clin Invest* 1992; **90:** 1122–1130.
5. Weiss J, Beckerdite-Quagliata S, Elsbach P. Determinants of the action of phospholipases A2 on the envelope phospholipids of *Escherichia coli*. *J Biol Chem* 1979; **254:** 11010–11014.
6. Forst S, Weiss J, Maraganoe JM, Heinrikson RL, Elsbach P. Relation between binding and the action of phospholipases A2 on *Escherichia coli* exposed to the bactericidal/permeability-increasing protein of neutrophils. *Biochem Biophys Acta* 1987; **920:** 221–225.
7. Wilde CG, Seilhamer JJ, McGrogan M, *et al.* Bactericidal/permeability-increasing protein and lipopolysaccharide (LPS)-binding protein. *J Biol Chem* 1994; **269:** 17411–17416.
8. Tobias PS, Ulevitch RJ. Lipopolysaccharide-binding-protein (LBP) and CD14 in lipopolysaccharide-dependent macrophage activation. *Immunobiol* 1993; **187:** 227–232.
9. Haziot A, Tsuberi BZ, Goyert SM. Neutrophil CD14: biochemical properties and role in the secretion of tumor necrosis factor-alpha in response to lipopolysaccharide. *J Immunol* 1993; **150:** 15556–15565.
10. Ischii Y, Wang Y, Haziot A, Del Vecchio PJ, Goyert SM, Malik AB. Lipopolysaccharide-binding-protein and CD14 interaction induces tumor necrosis factor-alpha generation and neutrophil sequestration in lungs after intratracheal endotoxin. *Canc Res* 1993; **73:** 15–23.
11. Heumann D, Gallay P, Betz-Corradin S, Barras C, Baumgartner JD, Glauser MP. Competition between bactericidal/permeability-increasing protein and lipopolysaccharide-binding-protein for binding to monocytes. *J Infect Dis* 1993; **167:** 1351–1357.
12. Dentener WA, van Asmuth EJ, Francot GJ, Marra MN, Buurman WA. Antagonistic effects of lipopolysaccharide-binding-protein and bactericidal/permeability-increasing protein on lipopolysaccharide (LPS) induced cytokine release by mononuclear phagocytes. Competition for binding to LPS. *J Immunol* 1993; **151:** 4258–4265.
13. Ooi CE, Weiss J, Doerfler ME, Elsbach P. Endotoxin neutralizing properties of the 25-kD N-terminal fragment and a newly isolated 30-kD C-terminal fragment of the 55-60 kD bactericidal/permeability-increasing protein of human neutrophils. *J Exp Med* 1991; **174:** 649–655.
14. Marra MN, Thornton MB, Snable JL, Wilde CG, Scott RW. Endotoxin binding and neutralizing properties of recombinant bactericidal/permeability-increasing protein and monoclonal antibodies HA-1A and E5. *Crit Care Med* 1994; **22:** 559–565.
15. Gazzano-Santoro H, Meszaros K, Birr C, *et al.* rBPI23, a recombinant fragment of bactericidal/permeability-increasing protein and lipopolysaccharide-binding-protein for binding to lipopolysaccharide and gram-negative bacteria. *Infect Immun* 1994; **62:** 1185–1191.
16. Corradin SB, Heumann D, Gallay P, Smith J, Manel J, Glauser MP. Bactericidal/permeability-increasing protein inhibits induction of macrophage nitric oxide production by lipopolysaccharide. *J Infect Dis* 1994; **169:** 105–111.
17. Ammons WS, Kung AH. Recombinant amino-terminal fragment of bactericidal/permeability-increasing protein prevents hemodynamic responses to endotoxin. *Circ Shock* 1993; **41:** 176–184.
18. Kohn FR, Ammons WS, Horwitz A, Grinman L, Theofan G, Weickmann J, Kung AH. Protective effect of a recombinant amino-terminal fragment of bactericidal/permeability-increasing protein in experimental endotoxemia. *J Infect Dis* 1993; **168:** 1307–1310.
19. Meszaros K, Parent JB, Gazzano-Santoro H, *et al.* A recombinant amino-terminal fragment of bactericidal/permeability-increasing protein inhibits the induction of leucocyte responses to lipopolysaccharide. *J Leukoc Biol* 1993; **54:** 558–563.
20. Fisher Jr CJ, Marra MN, Palardy JE, Marchbanks CR, Scott RW, Opal SM. Human neutrophil bactericidal/permeability-increasing protein reduces mortality rate from endotoxin challenge: a placebo-controlled study. *Crit Care Med* 1994; **22:** 553–558.
21. Gazzano-Santoro H, Parent JB, Grinman L, *et al.* High-affinity binding of the bactericidal/permeability-increasing protein and a recombinant amino-terminal fragment to the lipid-A region of lipopolysaccharide. *Infect Immun* 1992; **60:** 4754–4761.
22. Marra MN, Wilde CG, Collins MS, Snable JL, Thornton MB, Scott RW. The role of bactericidal/permeability-increasing protein as a natural inhibitor of bacterial endotoxin. *J Immunol* 1992; **148:** 532–537.
23. Weiss J, Wright G. Mobilization and function of extracellular phospholipase A2 in inflammation. *Adv Exp Med Biol* 1990; **275:** 103–113.
24. Tobias PS, Soldau K, Ulevitch RJ. Identification of a lipid A binding site in the acute phase reactant lipopolysaccharide-binding-protein (LBP). *J Biol Chem* 1989; **264:** 10867–10871.
25. Mathison J, Tobias P, Wolfson E, Ulevitch RJ. Regulatory mechanisms of host responsiveness to endotoxin (lipopolysaccharide). *Pathobiology* 1991; **59:** 185–188.
26. Wilde CG, Seilhamer M, McGrogan N, *et al.* Bactericidal/permeability-increasing protein and lipopolysaccharide (LPS)-binding protein. *J Biol Chem* 1994; **269:** 17411–17416.
27. Hsu SM, Raine L, Fanger H. The use of avidin–biotin–peroxidase complex (ABC) in immunoperoxidase techniques: a comparison between ABC and unlabeled antibody (PAP) procedures. *J Histochem Cytochem* 1981; **29:** 577–580.
28. White ML, Ma JK, Birr CA, Trown PW, Carroll SF. Measurement of bactericidal/permeability-increasing protein in human body fluids by sandwich ELISA. *J Immun Methods* 1994; **167:** 227–235.

ACKNOWLEDGEMENTS. The authors wish to thank Dr Marian Marra for recombinant human BPI and Ms Virpi Myllys for immunohistochemical preparations. This work was supported by Academy of Finland, the University of Turku Foundation, and Cancer Society of Southwestern Finland.

**Received 11 October 1995; accepted in revised form 8 December 1995**
